# BMC Veterinary Research reviewer acknowledgement, 2012

**DOI:** 10.1186/1746-6148-9-148

**Published:** 2013-07-26

**Authors:** Hayley Henderson

**Affiliations:** 1 BioMed Central, Floor 6, 236 Gray's Inn Road, London, WC1X 8HB, United Kingdom

## Abstract

**Contributing reviewers:**

The editors of *BMC Veterinary Research* would like to thank all our reviewers who have contributed to the journal in Volume 8 (2012).

## 

Jeffrey Abbott

USA

Najah Abi Gerges

United Kingdom

Zbigniew Adamiak

Poland

Isaiah Ademola

Nigeria

Amie Adkin

United Kingdom

Lis Alban

Denmark

Robyn Alders

Australia

Karin Allenspach

United Kingdom

Alberto Allepuz

Spain

Sandra Allweiler

USA

Sonia Almeria

Spain

Covadonga Alonso

Spain

Silvia Alonso

United Kingdom

Julio Alvarez

Spain

Flávio Ribeiro Alves Alves

Brazil

Sharif Aly

USA

Sandra Amass

USA

Carlos Ambrosio

Brazil

Tom Appleton

Canada

Shozo Arai

Japan

Toshiro Arai

Japan

Marie Archambault

Canada

Alberto Arencibia

Spain

David Argyle

United Kingdom

Pascal Arne

France

Stefan Arnhold

Germany

Mark Arnold

United Kingdom

Norma Arrigoni

Italy

Julie Arsenault

Canada

Lucy Asher

United Kingdom

Spiridoula Athanasiadou

United Kingdom

Ruben Avendaño-Herrera

Chile

Roger Ayling

United Kingdom

Osama Azawi

Iraq

Kibeom Bae

Korea, South

Wolfgang Baeumer

Germany

Yu Bai

China

Zoltan Bakos

United Kingdom

Anne Balkema-Buschmann

Germany

Elizabeth Ballegeer

USA

Nina Baltes

Germany

Isabel Bandín

Spain

Berit Bangoura

Germany

Barbara Bank-Wolf

Germany

John Bannantine

USA

Ashley Banyard

United Kingdom

Janice Barr

United Kingdom

Paul Barrow

United Kingdom

Joe Bartges

USA

Anna Bassols

Spain

Max Bastian

Germany

Wolfgang Baumgärtner

Germany

Frank Timo Beil

Germany

Elsa Beltran

United Kingdom

Cinzia Benazzi

Italy

Lisa Benjamin

USA

Lise C. Berg

Denmark

Dirk Berkvens

Belgium

Jean-Francois Bernardet

France

Eduardo Berriatua

Spain

Urban Besenfelder

Austria

Emma Best

United Kingdom

Hawthorne Beyer

United Kingdom

Giacomo Biagi

Italy

Tomasz Bielecki

Poland

Dorothee Bienzle

Canada

Charlotte Bjornvad

Denmark

Barbara Blacklaws

United Kingdom

Damer Blake

United Kingdom

Isabel Blanco Penedo

Sweden

Jose M Blasco

Spain

Jason Bleedorn

USA

Christian Blenn

Switzerland

Karen Blissitt

United Kingdom

Shauna Blois

Canada

Mariana Boadella

Spain

Peter Boettcher

Germany

Bojana Bogovic Matijasic

Slovenia

Lindsey Boone

USA

Michele Borgarelli

USA

Susanne Boroffka

Netherlands

Belen Borrego

Spain

Valeria Bortolaia

Denmark

Paolo Bosi

Italy

Corine Boucraut

France

Christoph Bourauel

Germany

Aniek Bouwman

Netherlands

Prosper Boyaka

USA

Elena Bozzetta

Italy

Vasco Branco

Portugal

Jackie Brearley

United Kingdom

Andrew Breed

United Kingdom

Walter Brehm

Germany

Paul Brett

USA

Tiziana A.L. Brevini

Italy

Stefano Brianza

Switzerland

Daniel Brown

USA

Glenn Browning

Australia

Sebastien Buczinski

Canada

Steven Budsberg

USA

Steven Budsberg

USA

Canio Buonavoglia

Italy

Graham Burgess

Australia

Patrick Butaye

Belgium

Simeon Cadmus

Nigeria

Kyle Caires

USA

Henrik Callesen

Denmark

Jacqui Calvin

United Kingdom

Bonnie Campbell

USA

Wenguang Cao

China

Tom Caperna

USA

Francesca Capolongo

Italy

Mariateresa Capucchio

Italy

James Carmalt

Australia

Adriano Carregaro

Brazil

Ana Carvajal

Spain

Jordi Casal

Spain

Geovanni Cassali

Brazil

Andy Catley

Ethiopia

Christophe Celeste

Canada

Pietro Celi

Australia

Natalia Cernicchiaro

USA

Jose Ceron

Spain

Mark Chambers

USA

Elizabeth Chan

United Kingdom

Jacky Chan

Taiwan

Pascale Chavatte-Palmer

France

Alberto O Chavez Velazquez

USA

Chau-Hwa Chi

Taiwan

Shau-Chi Chi

Taiwan

Tomasz Chmielewski

Poland

Jane Cho

USA

Bruno Chomel

USA

Chin Cheng Chou

Taiwan

Mette Christoffersen

Denmark

Rungtip Chuanchuen

Thailand

Jindrich Citek

Czech Republic

Tracy Clegg

Ireland

Dylan Clements

United Kingdom

Alison Clode

USA

Axel Cloeckaert

France

Lisa Collins

United Kingdom

Arianna Comin

Italy

Emmanuel Comoy

France

Andrew Conlan

United Kingdom

Franz Josef Conraths

Germany

Laurent Couetil

USA

Guillaume Counotte

Netherlands

Daniel Crocker

USA

Beate Crossley

USA

Attila Cságola

Hungary

Sally Cutler

United Kingdom

Joanna Cymerys

Poland

Sven Dänicke

Germany

Pierre-Yves Daoust

Canada

Kristiaan D'Août

Belgium

Arwid Daugschies

Germany

Rebecca Davidson

Norway

Damian F. De Andres

Spain

Aline De Koeijer

Netherlands

Geoffrey De Lisle

New Zealand

Hilde De Rooster

Belgium

Benoit De Thoisy

French Guiana

Victor Javier Del Rio Vilas

United Kingdom

Amy Delgado

USA

Odir Dellagostin

Brazil

Ilaria Di Bartolo

Italy

Barbara Di Martino

Italy

Ivan Diaz

Spain

Gillian Diesel

United Kingdom

Michael Dikeman

USA

Qian Ding

USA

Ramiro Dip

Switzerland

Sara Divari

Italy

Padraic Dixon

United Kingdom

Steven Djordjevic

Australia

Britta Dobenecker

Germany

Jose-Maria Dominguez-Roldan

Spain

Steven Dow

USA

Tomasz Drewa

Poland

Michele Drigo

Italy

Robert Drillien

France

Jozsef Dudas

Austria

Todd Duffield

Canada

Simon Dufour

Canada

Tanya Duke

Canada

Gilles Dupre

Austria

Susan Eades

USA

Iain East

Australia

C H Edinboro

USA

Tom Edrington

USA

Agneta Egenvall

Sweden

John Egerton

Australia

László Egyed

Hungary

Susan Eicher

USA

Abel Ekiri

USA

Meike Eklund

Germany

Chris Elliott

United Kingdom

Bas Engel

Netherlands

Gunes Erdogan

Turkey

Nuria Eritja

Spain

Kevin Esch

USA

Arild Espenes

Norway

John Fairbrother

Canada

Joseph Falkinham

USA

Thomas Famula

USA

Timothy Fan

USA

Colin Farquharson

United Kingdom

Vahab Farzan

Canada

Folorunso Oludayo Fasina

South Africa

Mujeeb Fazili

India

Christine Fehlner-Gardiner

Canada

Francis Fieni

France

**Johanna Fink**-**Gremmels**

Netherlands

Christopher Finnegan

United Kingdom

**Zlata Flegar**-**Mestric**

Croatia

Maria Aparecida A Koike Folgueira

Brazil

Jason Folster

USA

Leila Fonseca

Brazil

Maria Forzan

Canada

Caroline Fossum

Sweden

Leo Foyle

Australia

Lorenzo Fraile

Spain

Robert Friendship

Canada

**Jean**-**Pierre Frossard**

United Kingdom

**Yong**-**Fu Fu**

China

Walter Fuchs

Germany

Pablo Fuentes

Spain

Masayuki Funaba

Japan

Victor Gannon

Canada

Anapatricia Garcia

USA

**Ignacio Garcia**-**Bocanegra**

Spain

**Ana L. Garcia**-**Perez**

Spain

Ian Gardner

USA

Michael Gardner

USA

Fátima Gärtner

Portugal

Rony Geers

Belgium

Gabriele Gerardi

Italy

Wilhelm Gerner

Austria

Lutz Geue

Germany

Tracy Gieger

USA

Robert Gilbert

USA

Pedro J. Ginel

Spain

Erika Ginsburg

USA

Andrea Gioffre

Argentina

Liz Glass

United Kingdom

Giacomo Gnudi

Italy

Cengiz Gokbulut

Turkey

Vitor Goncalves

Brazil

Jose Luis Gonzales

Netherlands

Lorenzo Gonzalez

United Kingdom

Margaret Good

Ireland

Ira Gordon

USA

Christian Gortazar

Spain

Simon Graham

United Kingdom

Nicolas Granger

France

Laura Green

United Kingdom

Valeria Grieco

Italy

Sylvia Grierson

United Kingdom

Frank Griffin

New Zealand

Mirjam Grobbel

Germany

Andrea Gröne

Netherlands

Elisabeth Grosse Beilage

Germany

Edward Groth

USA

Christian Grund

Germany

Luca Guardabassi

Denmark

Sebastian Guenther

Germany

Panduka Gunawardena

Sri Lanka

Franco Guscetti

Switzerland

Amanda Guth

USA

Carlos Gutierrez

Spain

Silvina Gutiérrez

Argentina

Hansjoachim Hackbarth

Germany

Daniela Christina Hadorn

Switzerland

Freddy Haesebrouck

Belgium

Zhao Haifeng

China

Hayley Haining

United Kingdom

Gayle Hallowell

United Kingdom

Omar Hamarsheh

Palestinian Territory

Gawain Hammond

United Kingdom

**Tom Harcourt**-**Brown**

United Kingdom

Tara Harrison

USA

Daisuke Hasegawa

Japan

Marlene Hauck

USA

Martine Hausberger

France

Kevin Haussler

USA

Silke Hecht

USA

Jake Heiney

USA

Hege Hellberg

Norway

**Gro**-**Ingunn Hemre**

Norway

Wouter Hendriks

Netherlands

Diane Hendrix

USA

Pedro Herráez

Spain

Rodolphe Hervé

United Kingdom

Ann Hess

USA

**Anna Hielm**-**Bjorkman**

Finland

Yoshiaki Hikasa

Japan

Jf Hocquette

France

Douglas Hodgins

Canada

Ursula Hofle

Spain

**Regina Hofmann**-**Lehmann**

Switzerland

Merete Hofshagen

Norway

Casey Holliday

USA

Dietmar Holm

South Africa

Lindsey Holmstrom

USA

Jayne Hope

United Kingdom

Markus Horning

USA

Daniel Horton

United Kingdom

Katherine Houpt

USA

Mari Hovinen

Finland

Bjorn Hoyheim

Norway

Xiuguo Hua

China

Jin Huang

China

Yamamoto Ichiro

Japan

Isabelle Iff

Switzerland

Jo Ireland

United Kingdom

Vladimir Isachenko

Germany

Katsumi Ishioka

Japan

Reza Izadpanah

USA

Abdul Jabbar

Australia

Christine Jansen

Netherlands

Désirée Jansson

Sweden

Eva Jansson

Sweden

Thierry Jauniaux

Belgium

Nick Jeffery

USA

Vladimir Jekl

Czech Republic

Paul Jepson

United Kingdom

Katarina Jewgenow

Germany

Zhihua Jiang

USA

Ling Jin

USA

Anja Joachim

Austria

Roger Johnson

Canada

Wesley Johnson

USA

David Jordan

Australia

Ferran Jori

South Africa

Kamani Joshua

Nigeria

Véronique Julliand

France

Polona Juntes

Slovenia

Ramon A. Juste

Spain

Kristina Kadlec

Germany

John Kagira

Kenya

Josef Kamphues

Germany

Martin Kaske

Germany

Martin Kasny

Czech Republic

Philip Kass

USA

Bart Keijser

Netherlands

Gilbert Kersh

USA

Muhammad Khalid

United Kingdom

Pattara Khamrin

Thailand

Sangeeta Khare

USA

Linda Kidd

USA

Manfred Kietzmann

Germany

Gonhyung Kim

Korea South

**Shin**-**Hee Kim**

USA

Robert Kirberger

South Africa

Jolle Kirpensteijn

Netherlands

William Kisseberth

USA

Go Kitahara

Japan

Barbara Kitchell

USA

**Mads Kjelgaard**-**Hansen**

Denmark

Adam Kleczkowski

United Kingdom

Phillip Klesius

USA

Karen Kline

USA

Kirstine Klitgaard

Denmark

Gary Kobinger

Canada

Matt Koci

USA

Wolfgang Koester

Canada

Niels Komen

Belgium

Polychronis Kostoulas

Greece

Sabine Kramer

Germany

Erna Geessien Kroon

Brazil

Jens H. Kuhn

USA

Janina Kulka

Hungary

Subodh Kumar

India

Rajesh Kumar Vaid

India

Tetsuo Kunieda

Japan

Juan Kusanovic

USA

Tae Gyun Kwon

Korea, South

Grace Kwong

Canada

Jane Ladlow

United Kingdom

Monique Lafon

France

Christoph Lämmler

Germany

Margarita Lampo

Venezuela

**Sorrel Langley**-**Hobbs**

United Kingdom

Cristina Lanzas

USA

Sylvain Larrat

Canada

Michel Laurentie

France

**Jean**-**Pierre Lavoie**

Canada

**Anouk Lavoie**-**Lamoureux**

Canada

Richard Lea

United Kingdom

Lyon Lee

USA

Michael Lee

United Kingdom

Peter Leegwater

Netherlands

Alfred Legendre

USA

José Manuel Leiro Vidal

Spain

Britta Leise

USA

Caroline Leroux

France

Marie Lewis

United Kingdom

J Lievaart

Australia

Donald Lightner

USA

Casper Lindegaard

Sweden

Victoria Lipscomb

United Kingdom

Lihong Liu

Sweden

Sunny Liu

USA

Michael Livesey

USA

**Shyh**-**Ching Lo**

USA

Zelia Lobato

Brazil

**Jack "Mitchell**" **Lockhart**

USA

Brandon Londt

United Kingdom

Sam Long

Australia

Célia Lopes

Portugal

Emma Love

United Kingdom

Chengping Lu

China

Katharine Lunn

USA

Blanca Lupiani

USA

N James Maclachlan

USA

Adam Macneil

USA

Amy Macneill

USA

Dominiek Maes

Belgium

Bergljot Magnadottir

Iceland

Anita Mahadevan

India

Wilfried Mai

USA

Viktoria Majlathova

Slovakia

Birgit Makoschey

Netherlands

Ignasi Marco

Spain

Francois Maree

South Africa

Maria Luisa Marenzoni

Italy

**Carine Marie**-**Magdeleine**

Guadeloupe

Mariarosaria Marinaro

Italy

Celia Marr

United Kingdom

Vito Martella

Italy

Paolo Martelli

Italy

Juana Martin De Las Mulas

Spain

Salim Mattar

Colombia

John J. Maurer

USA

Sandro Mazzariol

Italy

Laura Mazzucco

Italy

Kathleen Mc Entee

Belgium

Jere Mcbride

USA

Dominic Mccafferty

United Kingdom

Lawrence Mcgill

USA

Colin Mcinnes

United Kingdom

James Mckean

USA

Trevelyan Mckinley

United Kingdom

James Mcnair

United Kingdom

As Mcneilly

United Kingdom

Els Meeusen

Australia

Marina Meli

Switzerland

Miguel Mellado

United Kingdom

Noelita Melo De Sousa

Belgium

Locksley Messam

USA

Kristen Messenger

USA

Irén Mikó

Hungary

Javier Millan

Spain

Gay Miller

USA

Laura Miller

USA

Patti Miller

USA

Stephen Miller

Canada

Sam Millet

Belgium

Thimios Mitsiadis

Switzerland

Keiko Miyadera

USA

Shusei Mizushima

Japan

Mariko Mochizuki

Japan

Jaime Modiano

USA

Jan Mol

Netherlands

Gustavo Monti

Chile

Azucena Mora

Spain

Sofia Morais

Spain

Maxim Moreau

Canada

Anabela Moreira

Portugal

Nobuko Mori

Japan

Hassan Mousavi Takami

Iran

Peter Msoffe

Tanzania

Ralf Mueller

Germany

Martha Mulks

USA

Cecilia Múller

Sweden

Jennifer Mumaw

USA

**Hetron Munang**'**Andu**

Norway

Atsushi Murai

Japan

Yuichi Murayama

Japan

Jack Murphy

Ireland

Kathy Murphy

USA

Alexander Murray

United Kingdom

Jane Murray

United Kingdom

Jo Murrell

United Kingdom

Hanspeter Naegeli

Switzerland

Miho Nagasawa

Japan

Béla Nagy

Hungary

Gabor Nagy

Hungary

Krisztina Nagy

Hungary

Lubna Nasir

United Kingdom

Quiniou Nathalie

France

Heiko Nathues

Germany

Manabu Nemoto

Japan

Stephan Neumann

Germany

Mandy Nevel

United Kingdom

Stephen Nickerson

USA

Soeren Saxmose Nielsen

Denmark

Hassan Nili

Iran

Thomas Nolan

USA

Steen Nordentoft

Denmark

Susan Novak

Canada

Vasileios Ntafis

Greece

Alejandro Nunez

United Kingdom

Daryl Nydam

USA

Stephanie Nykamp

Canada

**Robert O**'**Brien**

USA

**Solomon (Wole**) **Odemuyiwa**

USA

Anselmo Odeon

Argentina

Gerhard Oechtering

Germany

Anna Oevermann

Switzerland

Hayato Ohshima

Japan

**Francisco Olea**-**Popelka**

USA

Javier Oliveira Alvarez

Spain

John Elmerdahl Olsen

Denmark

Christine Olver

USA

Takenori Onaga

Japan

Maarten Oosterlinck

Belgium

Zeynep Osar Siva

Turkey

Maria Cristina Ossiprandi

Italy

Leigh Owens

Australia

Mark Oyama

USA

Juliano Paccez

South Africa

Dominique Paepe

Belgium

Saverio Paltrinieri

Italy

Nikolaos Panousis

Greece

Lysimachos Papazoglou

Greece

Mark G. Papich

USA

Dennilyn Parker

Canada

Sonal Patel

Norway

David Paton

United Kingdom

Abby Patterson

USA

**Janet Patterson**-**Kane**

United Kingdom

David Pearl

Canada

Edmund Peeler

United Kingdom

Simone Peletto

Italy

Dustin Pendell

USA

Andres Perez

USA

Daniel Perez

USA

Ruben Perez

Chile

Sandra Perez

Argentina

Adalberto Pérez De León

USA

Lyall Petrie

Canada

Rob Pettitt

United Kingdom

Thilo Pfau

United Kingdom

Dirk Pfeiffer

United Kingdom

Ute Philipp

Germany

Cesar Picado

Spain

Jacqueline Picard

Australia

Giuseppe Piccione

Italy

Sofie Piepers

Belgium

Neville Pillay

South Africa

Gina Pinchbeck

United Kingdom

Robert Pirie

United Kingdom

Jeffrey Plowman

New Zealand

Patrícia Poeta

Portugal

Mark Pokras

USA

Alessandro Poli

Italy

Zvonimir Poljak

Canada

Gerry Polton

United Kingdom

Luc Poncelet

Belgium

Cornelis Poppe

Canada

Simon Pot

Switzerland

Christine Pourcel

France

Antonio Pozzi

USA

Abani Pradhan

USA

Cinta Prieto

Spain

Auriol Purdie

Australia

Satu Pyörälä

Finland

Bruno Pypendop

USA

F. Luisa Queiroga

Portugal

Heidi Radke

United Kingdom

De Maria Raffaella

Italy

Sheikh Raisuddin

India

Swaraj Rajkhowa

India

Alejandro Ramirez

USA

Antonio Ramis

Spain

Robert Rebhun

USA

Sergio Recuenco

USA

Erin Elizabeth Rees

Canada

Michael Reichel

Australia

Rudolf Reichel

United Kingdom

Scott Reid

United Kingdom

Olav Reksen

Norway

Jesus Requena

Spain

Ali Rezakhani

Iran

Anne Lisa Ridler

New Zealand

Irmgard Riedmaier

Germany

David Riley

USA

Barbara Riond

Switzerland

Robert Risebrough

USA

Giuseppe Rizzo

Italy

Sheilah Robertson

USA

Maria Rita Rodrigues

Brazil

Carlos Rodriguez

USA

Fernando Rodriguez

Spain

Mariarita Romanucci

Italy

Stefano Romussi

Italy

Helene Rosenberg

USA

Sophie Rossi

France

David Rowlands

United Kingdom

Nadia Rucci

Italy

Pamela Ruegg

USA

**Francisco Ruiz**-**Fons**

Spain

Jonathan Rushton

United Kingdom

Gerard Rutteman

Netherlands

Helena Rylander

USA

Simo Saarakkala

Finland

Heinz Sager

Switzerland

Afrah Salama

Egypt

Francisco Javier Salguero

United Kingdom

Charlotte Sandersen

Belgium

Yongming Sang

USA

Brandon Santoni

USA

Marcos Veiga Santos

Brazil

Giuseppe Sarli

Italy

Nobuo Sasaki

Japan

Mamoru Satoh

Japan

Jimmy Saunders

Belgium

Gereon Schares

Germany

Martin Schmidt

Germany

J Schrickx

Netherlands

Linda Schuler

USA

Herbert Schweizer

USA

Harvey Morgan Scott

USA

Peter Scrivani

USA

Julian Seago

United Kingdom

Hiroshi Sentsui

Japan

Kyoung Won Seo

Korea South

Torsten Seuberlich

Switzerland

Chris Seymour

United Kingdom

Devendra Shah

USA

Darren Shaw

United Kingdom

I Martin Sheldon

United Kingdom

G. Diane Shelton

USA

Workineh Shibeshi

Ethiopia

Nagini Siddavaram

India

**Karim Sidi**-**Boumedine**

France

Ursula Siebert

Germany

Hubert Simhofer

Austria

Heather Simmons

USA

Hugh Simmons

United Kingdom

Jane Sinclair

New Zealand

Rosa Sirianni

Italy

Francesca Sisto

Italy

Eystein Skjerve

Norway

Katherine Skorupski

USA

Joan Smyth

USA

Ricardo Soares Magalhaes

Australia

Mette Aamand Soerensen

Denmark

Sandra Solaiman

USA

Laura Soler Vasco

Belgium

Robert Somerville

United Kingdom

Daesub Song

Korea South

Glenn Songer

USA

Ulrike Sorge

USA

Iryna Sorokulova

USA

Carlos José Sousa Passos

Brazil

Fernando Souza

Brazil

Claudia Spadavecchia

Switzerland

Bernhard Spiess

Switzerland

Richard Squires

Australia

Frederic Stachurski

Madagascar

Kevin Stafford

New Zealand

Jan Stagsted

Denmark

Gavin Staley

USA

Amy L Stanton

USA

Elke Starick

Germany

Christoph Staubach

Germany

Paulo Steagall

Canada

Annalisa Stefani

Italy

Helle Stege

Denmark

Timothy Stein

USA

Falko Steinbach

United Kingdom

Adolf Steinrigl

Austria

Joanne Stevens

United Kingdom

Mark Stevenson

New Zealand

Allison Stewart

USA

Ulrich Stock

Germany

Karl Stoffel

Switzerland

Alistair Stott

United Kingdom

George Strain

USA

Sam Strain

United Kingdom

Erin Strait

USA

Lesley Stringer

New Zealand

Snorre Stuen

Norway

Jianguo Su

China

Subbaya Subramanian

USA

Martin Sullivan

United Kingdom

Kazuyuki Suzuki

Japan

Tohru Suzuki

Japan

Kendall Swanson

USA

Camille Szmaragd

United Kingdom

Sabine Tacke

Germany

**Abdessamad Tahiri**-**Alaoui**

United Kingdom

Willem Takken

Netherlands

Jesus Talavera

Spain

Amin Tamadon

Iran

Chen Tan

China

Veronika Tarageová

Slovakia

Mehri Tavakol

Netherlands

Nick Taylor

United Kingdom

Hiroyuki Tazaki

Japan

**Bernd**-**Alois Tenhagen**

Germany

Erik Teske

Netherlands

Caroline Tessier

France

Jens Tetens

Germany

Alana M Thackray

United Kingdom

Francois Thiaucourt

France

Simon Thierry

France

Etienne Thiry

Belgium

John Thomason

USA

Peter Thompson

South Africa

Kevin Thorneloe

USA

Barbara Thur

Switzerland

Andrea Tipold

Germany

Michael Tivers

United Kingdom

Nils Toft

Denmark

**Jenny**-**Ann Toribio**

Australia

**J. Felipe De J. Torres**-**Acosta**

Mexico

Sigurbjörg Torsteinsdottir

Iceland

Philippe Totte

France

**Pierre**-**Louis Toutain**

France

Bill Tranter

Australia

Henry Tremaine

United Kingdom

Erminio Trevisi

Italy

Eric Troncy

Canada

Karina Trono

Argentina

Anne Turner

Australia

Hermann Unger

Austria

Åse Uttenthal

Denmark

Francisco Uzal

USA

Outi Vainio

Finland

Eric Van Breda

Netherlands

Henri Van Bree

Belgium

Bart Van Den Borne

Switzerland

Hendrika Anette Van Dorland

Switzerland

Cornelis Van Elk

Netherlands

Ed Van Klink

United Kingdom

Anne Van Langendonckt

Belgium

Wannes Vanderhaeghen

Belgium

An Vanhaesebrouck

United Kingdom

Fabio Vannucci

USA

Agueda Vargas

Brazil

Estelle Venter

South Africa

Nuria Verdaguer

Spain

Kurt Verkest

Australia

**David Verner**-**Jeffreys**

United Kingdom

Frank Verstraete

USA

Lonneke Vervelde

Netherlands

Gesualdo Vesco

Italy

Lene Karine Vestby

Norway

Joaquin Vicente

Spain

Natassia Vieira

USA

Balamurugan Vinayagamurthy

India

Szilvia Vincze

Germany

Kathalijne Visser

Netherlands

Holger Volk

United Kingdom

Henrik Von Euler

Sweden

**Georg Von Samson**-**Himmelstjerna**

Germany

Wilna Vosloo

Australia

John Wallace

United Kingdom

Gemma Walmsley

United Kingdom

Birgit Walther

Germany

Chunlai Wang

China

Jinbao Wang

China

Alastair Ward

United Kingdom

John Wardale

United Kingdom

Sean Wattegedera

United Kingdom

Cynthia Webster

USA

Scott Weese

Canada

Zuzhang Wei

China

**Hsin**-**Yi Weng**

USA

Arno Werners

Grenada

Elias Westermarck

Finland

Lothar H. Wieler

Germany

Pam Wiener

United Kingdom

Bruce Wilkie

Canada

Jacintha Wilmink

Netherlands

Brenda Anne Wilson

USA

Henk Wisselink

Netherlands

Thomas Witte

United Kingdom

Moges Woldemeskel

USA

Shona Wood

United Kingdom

Peter Woolcock

USA

Qingmei Xie

China

Fei Xue

China

Kiyoshi Yamaguchi

Japan

Osamu Yamato

Japan

Dustin Yates

USA

Carl Yeoman

USA

Takashi Yokoyama

Japan

Donald Yool

United Kingdom

Yukinori Yoshimura

Japan

Dionisios Youlatos

Greece

Jesse Young

USA

Shuyang Yu

USA

Sanaa Zaki

Australia

Gianluigi Zanusso

Italy

Jürgen Zentek

Germany

Chao Fan Zhang

China

Min Zhao

USA

Rui Zhou

China

Enrica Zucca

Italy

Allison Zwingenberger

USA

